# Synthesis and Performance Evaluation of a Novel Nanoparticle Coupling Expanded Granule Plugging Agent

**DOI:** 10.3390/gels9060479

**Published:** 2023-06-12

**Authors:** Xuejiao Li, Qi Li, Meilong Fu, Li Li, Lingyang Su, Yingyang Wang

**Affiliations:** 1 Hubei Key Laboratory for Processing and Application of Catalytic Materials, College of Chemistry and Chemical Engineering, Huanggang Normal University, Huanggang 438000, China; 15949506481@163.com (Q.L.); 13227313655@163.com (L.L.); m2066503389@163.com (L.S.); wyy2309704308@163.com (Y.W.); 2 College of Petroleum Engineering, Yangtze University, Wuhan 430100, China; fml202208@163.com

**Keywords:** temperature and salt resistant type, expanded granule, nanoparticle SiO_2_, coupling, performance evaluation

## Abstract

This study focuses on the characteristics of fractured and vuggy high-temperature and high-salt reservoirs in the Tahe Oilfield. The Acrylamide/2-acrylamide-2-methylpropanesulfonic copolymer salt was selected as a polymer; the hydroquinone and hexamethylene tetramine was selected as the crosslinking agent with a ratio of 1:1; the nanoparticle SiO_2_ was selected, and its dosage was optimized to 0.3%; Additionally, a novel nanoparticle coupling polymer gel was independently synthesized. The surface of the gel was a three-dimensional network structure, with grids arranged in pieces and interlaced with each other, and the structure was very stable. The SiO_2_ nanoparticles were attached to the gel skeleton, forming effective coupling and enhancing the strength of the gel skeleton. To solve the problem of complex gel preparation and transportation, the novel gel is compressed, pelletized, and dried into expanded particles through industrial granulation, and the disadvantage of the rapid expansion of expanded particles is optimized through physical film coating treatment. Finally, a novel nanoparticle coupling expanded granule plugging agent was developed. Evaluation of the performance of the novel nanoparticle coupling expanded granule plugging agent. With an increase in temperature and mineralization, the expansion multiplier of granules decreases; aged under high-temperature and high-salt conditions for 30 days, the expansion multiplier of granules can still reach 3.5 times, the toughness index is 1.61, and the long-term stability of the granules can be good; the water plugging rate of granules is 97.84%, which is superior to other widely used particle-based plugging agents.

## 1. Introduction

The Tahe Oilfield is located in the Tarim Basin, a rare large ultra-deep marine seam-hole-type carbonate reservoir, with a planar well-controlled oil-bearing area of 2800 km^2^. By August 2019, the Tahe Oilfield submitted a total of 13.5 × 10^8^ t of proven petroleum geological reserves, and the cumulative oil production exceeded 1 × 10^8^ t. The Tahe Oilfield reservoirs are divided into fracture, seam-hole, and cavern. The reservoir temperature is as high as 120–150 °C, and the total mineralization of the formation water is (200–250) g/L, which belongs to the super high temperature and high salt reservoir [1,2]. At present, the main challenges encountered in the extraction process of the Tahe Oilfield are the rapid increase in water content of oil wells, rapid production reduction of oil wells, and overall deviation of the water plugging effect; therefore, it is urgent to slow down the increasing rate of water content after water is seen in oil wells [3].

A granular plugging agent is a popular plugging agent commonly used in water plugging and dissection. It is stable, has a wide variety, has good mechanical properties, and is suitable for plugging water in oil reservoirs with large pores. Expanded granules are a type of granular plugging agent developed in the last 20 years and can be expanded when encountering water; they are cross-linked, granulated, and dried on the ground, avoiding the influence of complex conditions of the formation on the cross-linking of the system, which can better plug the reservoir with large pores [4]. Compared with cement-based inorganic granular plugging agents and curable granular plugging agents widely used in the Tahe Oilfield, the expanded granules can absorb water and swell, can plug the throat with their deformation, or penetrate the formation through deformation under certain pressure differences to seal the high-permeability layer containing seams and, large pores [5,6].

In this paper, a novel nanoparticle coupling polymer gel was independently synthesized. The novel gel is compressed, pelletized, and dried into expanded particles through industrial granulation. The disadvantage of too fast expanded particle expansion is optimized through physical film coating treatment. Finally, a novel nanoparticle coupling expanded granule-plugging agent was developed. The novel gel was chemically characterized by FT-IR analysis, SEM, and a structural diagram, and the performance of the plugging agent was evaluated from the aspects of temperature impact on expansion ratio, salinity impact on expansion ratio, and long-term thermal stability of particles. This article provides favorable technical support for applying the bulk expanded granule plugging agent in the Tahe Oilfield.

## 2. Results and Discussions

### 2.1. Synthesis of Novel Nanoparticle Coupling Polymer Gel

#### 2.1.1. Polymerization of Polymer Gel

In this paper, Acrylamide/2-acrylamide-2-methyl propane sulfonic copolymer salt (AM/AMPS) was selected as the polymer. The AM/AMPS polymer was polymerized by acrylamide (AM) monomer and AMPS monomer; for AMPS monomer, the highly stable carbon chain was its main chain, and strong anionic hydration groups, i.e., methyl propyl sulfonate groups, were introduced into its molecule, which not only improved the hydrophilicity, but also greatly improved the salinity resistance of AMPS monomer due to the hyposensitivity of propane sulfonic acid groups to external cations; after the polymerization of acrylamide and AMPS monomer, the steric hindrance of polymer molecule significantly increased, effectively inhibiting the hydrolysis of amide groups, empowering strong stability to the polymer, and greatly improving its heat resistance and salt tolerance, which laid a solid foundation for the subsequent preparation of heat-resistant and salt-tolerant gels.

The hydroquinone and hexamethylenetetramine were selected as crosslinking agents with a ratio of 1:1. Hydroquinone and hexamethylenetetramine can form water-soluble (methyl) phenolic resin, which contains a benzene ring structure, making its temperature resistance even better; In addition, hydroquinone contains two hydroxyl groups. The ortho and para positions of the hydroxyl groups are chemical reaction active points, which is easier to form free hydroxymethyl groups so that they can be better crosslinked with AM/AMPS polymers, forming a network-like gel structure with a skeleton structure extending to four aspects.

**Figure 1:**
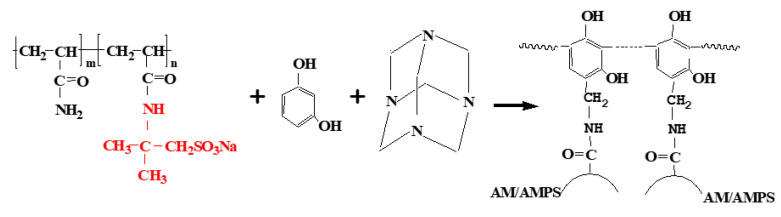
Schematic Diagram of Gel Structure.

The synthesis idea of polymer gel polymerization is as follows Figure 1:

#### 2.1.2. Screening and Dosage Optimization of Nanoparticles

The size range of nanoparticles is generally 0.1–100 nm. Nanoparticles coupled with polymer gel can strengthen the viscoelasticity and rheological properties of the gel system, enhance the strength of the gel skeleton, improve the water retention capacity, and form a higher strength and more compact filling network [7].

Nine different types of nanoparticles were screened out and added to the gel system. The viscosity and dehydration rate of the gel prepared with the Tahe simulated formation water at 130 °C in 20 days were investigated. The viscosity of the gel was measured by DV2T Viscometer, and the shear rate is 5.8 rpm. The experimental results are shown in Table 1.

According to Table 1, adding nanoparticles to the gel system can effectively improve the long-term stability of the gel system. The nanoparticle SiO_2_ was aged for 20 days under high temperatures and high salt, the viscosity of the gel was up to 43 Pa·s, and the dehydration rate was only 2.8%. Therefore, nano SiO_2_ was screened as coupling nanoparticles.

**Figure 2:**
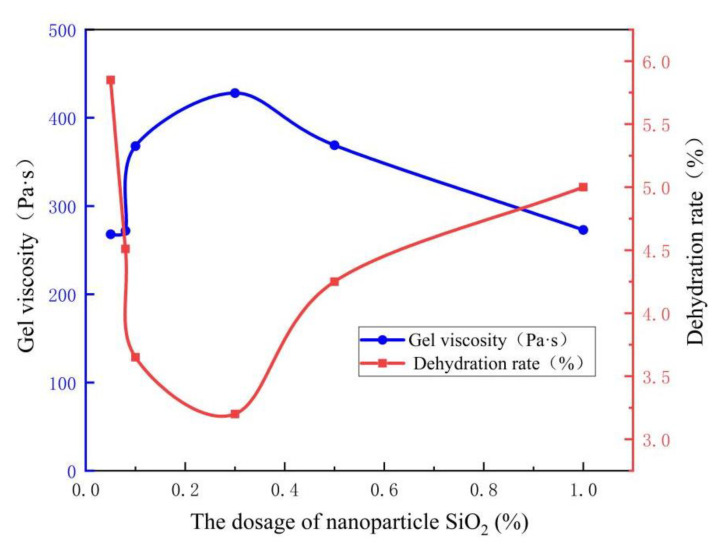
Optimization experiment of nanoparticle SiO_2_ dosage.

The dosage of nanoparticles was optimized by setting the dosage of nanoparticle SiO_2_ as 0.05%, 0.08%, 0.1%, 0.3%, 0.5%, and 1.0%, and other conditions remain unchanged. Using the Tahe simulated formation water to prepare the gel, and place the system at 130 °C for cross-linking reaction. The investigation time is 10 days. The experimental results are shown in Figure 2.

As shown in Figure 2, within a certain concentration range, the gelling strength of the system gradually increases with the increase of the dosage of nanoparticle SiO_2_. When the dosage of nanoparticle SiO_2_ reaches 0.3%, the gel viscosity is up to 42.8 mPa·s, and the dehydration rate for 10 days is only 3.2%. If the dosage of nanoparticle SiO_2_ continues to increase, the gelling strength of the system shows a downward trend, So 0.3% is the optimal dosage of nanoparticles.

**Figure 3:**
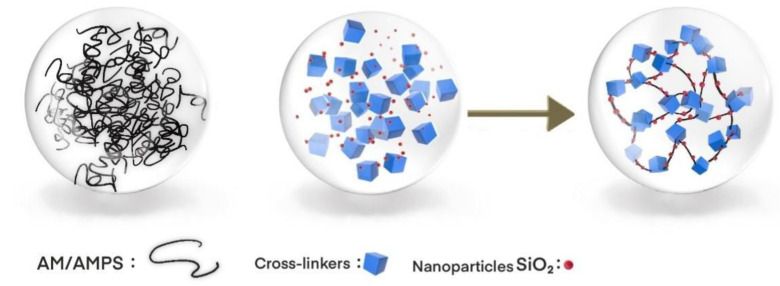
Structural Diagram of Nanoparticle Coupling Polymer Gel.

Nanoparticles have a surface effect, volume effect, quantum size effect, and macro quantum tunnel effect [8] and do not participate in chemical crosslinking reactions in the whole gel system. Nanoparticles are mainly combined with gel molecular chains in the way of hydrogen bonds, forming effective coupling, increasing the bonding point of the gel system, improving the tensile and compressive strength of the system, and greatly improving the stability of the gel system structure, The three-dimensional network skeleton of the gel system is effectively strengthened. In addition, nanoparticles coupled with polymer gel can effectively change the wettability of rock surfaces, reduce the interfacial tension between oil and water, and improve oil recovery efficiency. As shown in Figure 3.

#### 2.1.3. Chemical Structure Characterization of Nanoparticle Coupling Polymer Gel

**Figure 4:**
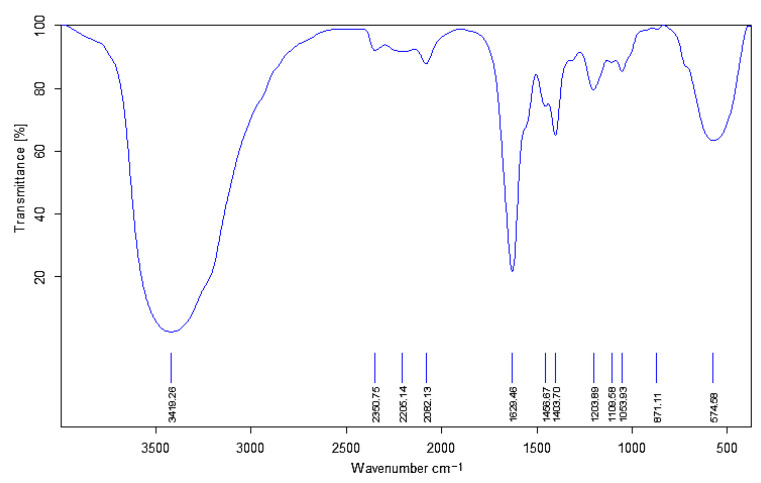
FT-IR analysis of nanoparticle coupled polymer gel (the concentration of SiO_2_ is 0.3%).

The nanoparticle coupling polymer gel belongs to soft-solid-like systems with a permanent system of bonds, which has high strength. According to FT-IR analysis, as shown in Figure 4. There is an obvious vibration absorption peak at 1203 cm^−1^, which belongs to the C-H vibration absorption peak in the benzene ring, which indicates that the aromatic ring crosslinker has been successfully introduced into the gel structure [9]. At the same time, under normal circumstances, the vibrational absorption peak of C=O in the amide group is at 1600 cm^−1^. Still, the vibrational absorption peak of C=O in the amide group in this graph moves to 1629 cm^−1^, with a significant shift, indicating that the amide group in AM/AMPS undergoes a crosslinking reaction with the crosslinking agent [10].

**Figure 5:**
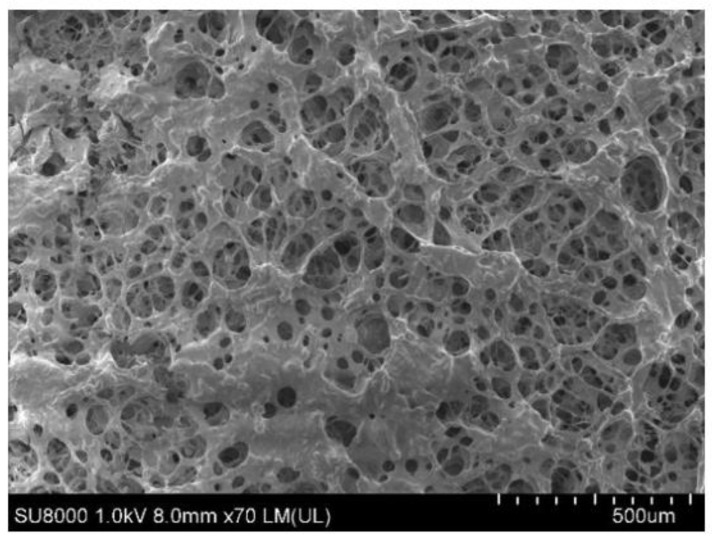
SEM of nanoparticle coupling polymer gel (the concentration of SiO_2_ is 0.3%).

The microstructure of the nanoparticle coupling polymer gel was analyzed by SEM, as shown in Figure 5.

As shown in Figure 5, the surface of the novel nanoparticle coupling polymer gel is a three-dimensional network structure, with grids arranged in pieces and interlaced with each other, and the structure is very stable.

### 2.2. Preparation of Laminated Expanded Granules

**Figure 6:**
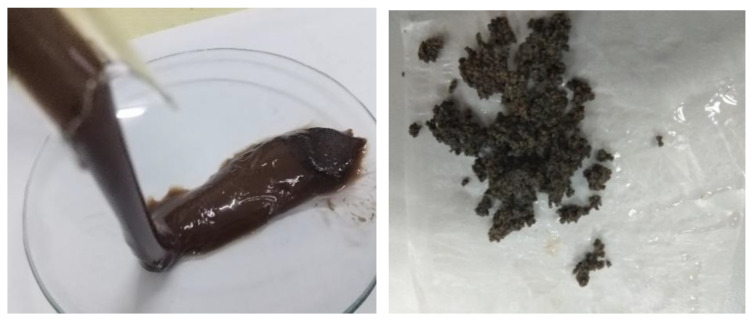
Morphology of nanoparticle coupling polymer gel (**left**) and nanoparticle coupling expansion granules (**right**).

The new nanoparticle coupled polymer gel has a stable three-dimensional network structure, good temperature, and salt resistance, and it is suitable for water plugging and profile control of fracture cavity reservoirs in high temperature and high salinity reservoirs in Tahe Oilfield. However, the preparation of the polymer gel is complex, and the transportation is tedious. To facilitate the on-site construction, the polymer gel is processed, compressed, pelletized, and dried into nanoparticle coupling expansion granules, as shown in Figure 6.

The novel expansion granules have been treated professionally in industrialization, and the gel framework structure has been preserved. In a reservoir with rich water content, the granules can absorb water and expand. This is due to the main chain of the granule molecule containing more hydrophilic groups. When the granule first comes into contact with water, the hydrophilic functional group can hydrate with water and draw water molecules into the interior of the granule. The water molecules can form hydrogen bonds with the main chain of the granule molecule, making water enter the granule molecule’s three-dimensional network structure smoothly. The granule network-like structure is very stable and has a good water retention capacity, which makes the osmotic pressure difference formed inside and outside the granule molecule. Under this pressure difference, the granules can be induced to absorb water continuously until the dissolution equilibrium is reached inside and outside the molecule [11]. According to the whole aspect, the granule water absorption swelling results from both physical and chemical adsorption. The absorbed water exists in the network structure mainly in two states: free water molecules and hydrated molecules formed by hydrogen bonds. 

The expansion rate of the nanoparticle coupling expansion granule is too fast. The expansion multiplier can reach 4.7 times within 0.5 h in the Tahe simulated stratigraphic water. The maximum expansion multiplier is reached within 8 h, which badly affects the injection of field granules. The novel expansion granules were optimized for achieving a slow expansion effect.

**Figure 7:**
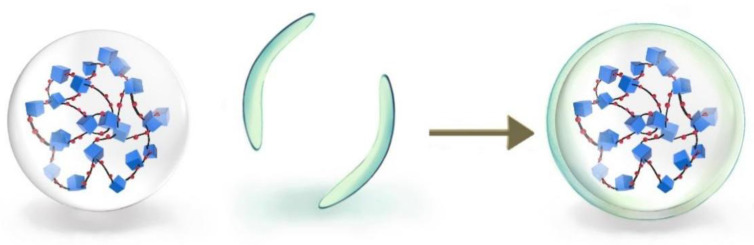
Structural diagram of overcoated expanded granules.

This section exhibits the idea of physical coating of the granules by using reagents that do not react chemically with the granules [12] to form a coating film on its surface, thus retarding its expansion, as shown in Figure 7. Polyvinylidene chloride viscosity (average molecular weight of 3 million), unsaturated sulfide (antioxidant), and organophosphate (stabilizer) were used to formulate the laminating solution. Polyvinylidene chloride has strong intermolecular cohesiveness, high crystallinity, excellent barrier properties, and does not react chemically with the granules. It can well physically coat the granules and form a capsule-like coating on their surface, thus, the swelling gets retarding. 

The 1% and 2% coating solutions were prepared in the laboratory, and the nanoparticle coupling expansion granules were coated with a film. Finally, two types of coating granules were prepared, which were the expanded granules with a film and the expanded granules with a thick film.

**Figure 8:**
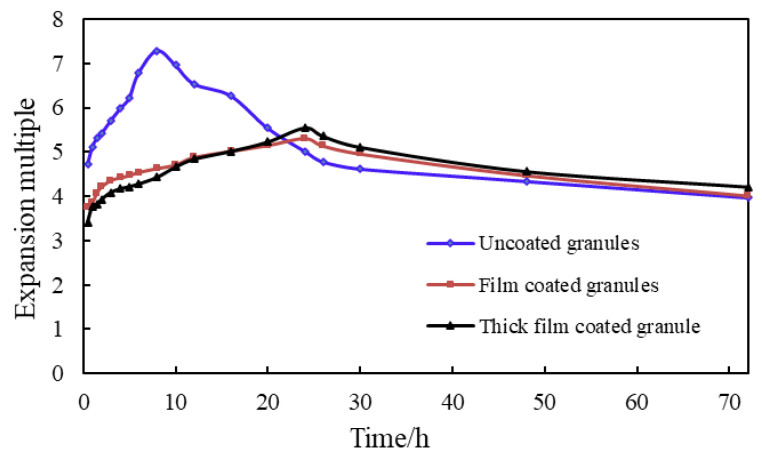
Evaluation Experiment of Expansion Performance of Overcoated Expanded Granules.

According to Figure 8, the initial expansion of the granule is rapid, reaching the maximum expansion multiplier within 8 h. In contrast, the thin and thick film of the granule begins to slowly decompose at high temperatures, and the granule slowly expands, reaching the maximum expansion multiplier of 5.31 within about 24 h, with good slow expansion performance. After the granules reached the maximum expansion multiplier, the granule began to shrink slowly and finally stabilized gradually, the expansion multiplier of 72 h stabilized at about 4 times.

Considering the effect of retarding expansion, economic costs, and other factors, using thin overcoated expanded granules is recommended.

### 2.3. Evaluation of the Nanoparticle Coupling Expanded Granule

#### 2.3.1. Effect of Temperature on the Expansion Multiplier

**Figure 9:**
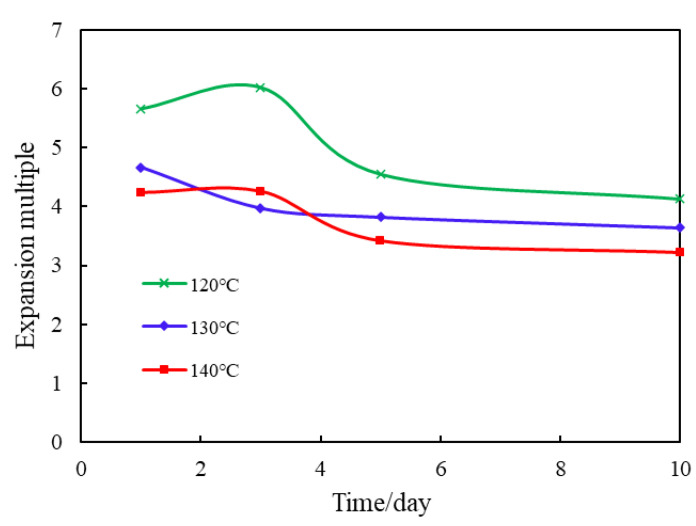
Effect of Temperature on the Expansion Multiplier of the Laminated Expanded Granules.

There is a test to the effect of temperature on the expansion multiplier of overcoated granules, Tahe Oilfield is a high-temperature reservoir, so three higher temperature gradients, 120 °C, 130 °C, and 140 °C, are set. By comparing the expansion performance of overcoated type expansion granules in 1–10 days, the experimental results are shown in Figure 9.

**Figure 10:**
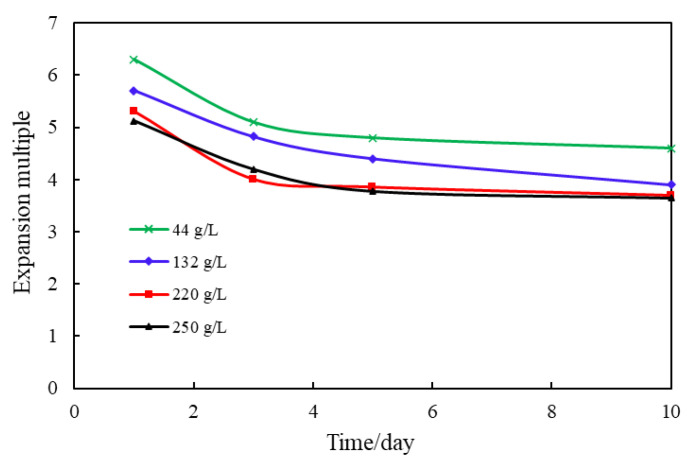
Effect of mineralization on the expansion multiplier of the laminated expanded granules.

According to Figure 10: the granule swelling multiplier of 1 day is 5.66 under 120 °C condition, and the swelling multiplier of 10 days can reach 4.13. Under the condition of 140 °C, the granule swelling multiplier declines to 4.23 after 1 day, and the swelling multiplier after 10 days is 3.21. The granule swelling multiplier is between the above temperatures under 130 °C. It shows that the temperature has a great influence on the granule swelling multiplier, and the granule swelling multiplier tends to decrease as the temperature increases. The results indicate that the high temperature reduces the performance of water-absorbing functional groups on the surface of the granules, the water molecules entering the molecular network structure of the granules are reduced, which has a certain inhibitory effect on the water absorption capacity of the granules [13,14].

#### 2.3.2. Effect of Mineralization Degree on Expansion Multiplier

The mineralization of the simulated formation water in Tahe River was 220 g/L. The simulated formation water was diluted with distilled water by 20% and 60%, and the water samples with mineralization of 44 g/L and 132 g/L were prepared, respectively. 3% NaCl was added to the simulated formation water and water samples with mineralization of 250 g/L were prepared to investigate the changes in granule swelling times under different mineralization. The evaluation time was 10 days and the experimental results are shown in Figure 10.

From the figure, we could know that when the mineralization of the water sample rises from 44 g/L to 220 g/L, the swelling multiplier of the granules shows an obvious decreasing trend. There are several main reasons for this. First, according to the theory of absorption thermo dynamics, the size of the ionic strength of the external solution can affect the absorption capacity of the body’s expansion particles. With the increase of mineralization, the ionic strength in the solution increases. The osmotic pressure inside and outside the granules’ molecules also decreases, inhibiting the expanded granules’ water absorption capacity, and the swelling multiplier decreases accordingly. In addition, the Ca^2+^ ion content in the Tahe model formation water is high, and Ca^2+^ ions affect the functional groups’ activity on the granule macromolecules’ surface, making the swelling multiplier decrease. When the mineralization of the water sample increased from 220 g/L to 250 g/L, the change in the swelling multiplier was very small, indicating that the change in the granule swelling multiplier decreased when the mineralization exceeded a certain range [15].

#### 2.3.3. Long-Term Thermal Stability of the Granules

The 7 groups of granule suspensions of the same granule size and 10% mass concentration were prepared with simulated formation water (mineralization of 220 g/L) from the Tahe River and placed at 130 °C for 30 days. They were removed at 2 h, 1 day, 2 days, 5 days, 10 days, 20 days, and 30 days. The granule swelling multiples and the granule strength were measured to evaluate the long-term stability performance of the granules under high temperatures and high salt.

Here, J. E. Smith’s toughness index method was used to measure the strength of the granules, quantifying the tensile and compressive strength of the granules. The toughness index can be defined as follows: selecting a sieve with a suitable mesh (the mesh should be smaller than the granule size after expansion), and measuring the pressure difference between the granules passing through the sieve twice (P1, P2). The multiplier of the two pressure differences is the toughness index, that is toughness index = P1/P2, the closer the toughness index is to 1.0, the better the strength of the granules. The pressure difference between the granules passing through the screen twice before and after different aging times was recorded and the toughness index value was calculated, as shown in Table 2.

**Figure 11:**
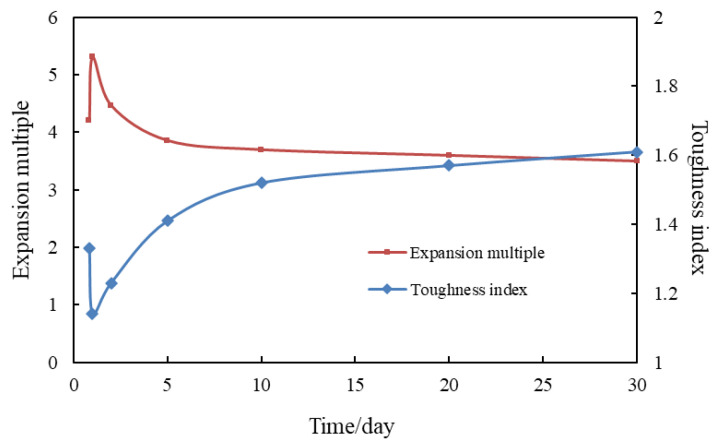
Experiment to evaluate the long-term stability performance of the granules.

To summarize and compare the experimental results of the expansion multiplier and toughness index measured by aging the granules under high temperature and high salt for 2 h–30 days, as shown in Figure 11.

According to Figure 11, under the conditions of high temperature and high salt, the granules swell slowly from 0 to 1 day, and the swelling multiplier increases linearly, while the toughness index decreases, reaching the maximum swelling multiplier of 5.31 and the minimum toughness index 1.14 within 1 day. This indicates that the granule strength increases with the increase of the swelling multiplier during the water absorption stage, the granule swelling multiplier and strength both reach the maximum in 1 day. This is mainly due to the high temperature and high salt, which have a certain inhibitory effect on the granules’ water absorption capacity and surface functional group activity [16]. After 5 days, the curves of the expansion multiplier and toughness index all tend to be stable, and after 30 days of aging, the expansion multiplier of the granules can reach 3.5 times. The toughness index goes to 1.61, which can meet the water plugging requirements of the Tahe Oilfield, indicating that the granules have good long-term stability under high temperature and high salt conditions.

#### 2.3.4. Granule Sealing Reservoir Performance

**Figure 12:**
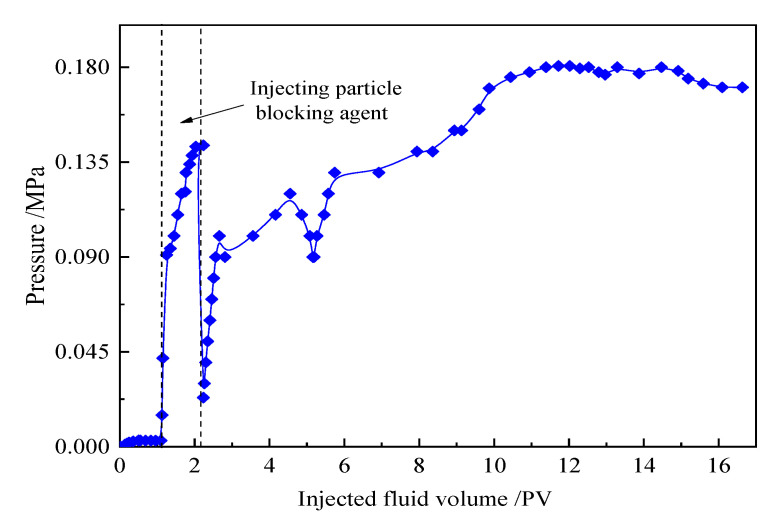
Pressure variation curve of core flow experiment.

The core flow experiments were conducted to evaluate the water-blocking ability of the granules in the reservoir. In the experiment, the saturated oil was dehydrated, and degassed crude oil from Tahe Oilfield (with a viscosity of 55.1 mPa·s), and the injected water was simulated with Tahe formation water at an injection rate of 0.5 mL/min, as shown in Figure 12.

**Figure 13:**
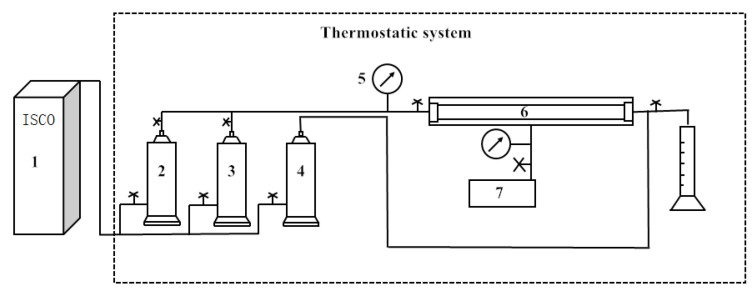
Core flow experimental device. 1- High pressure plunger pump; 2- Simulated oil; 3- Formation water; 4- Plugging agent solution; 5- Pres sure gauge; 6- Core gripper; 7- Hand pump.

The particles slowly absorb water and expand in the cracks and pores, and the expanded particles are prone to accumulate and bridge at the junction of the cracks and pores, forming a sealing effect. Based on the pressure difference data in Figure 13, the water phase permeability K_w_ before particle injection and the water phase permeability Kw after injection of the sealing agent is calculated; the particle water blocking rate is Φ_W_ = (K_w_ − K_w′_)/K_w_ × 100% = 97.84%. It can be seen that particles can effectively seal cracks and holes, with a sealing rate superior to other widely used particle-based plugging agents.

## 3. Conclusions

1. This article focuses on high-temperature and high-salt reservoirs in the Tahe Oilfield. Selecting Acrylamide/2-acrylamido-2-methylpropane sulfonic acid copolymer as a polymer; selecting hydroquinone and hexamethylene tetramine as the crosslinking agent with a ratio of 1:1. To enhance the stability of the system, nanoparticles are added to the system. The nanoparticle SiO_2_ was selected from nine different types of nanoparticles, and its dosage was optimized to 0.3%; A novel nanoparticle coupling polymer gel was synthesized.
2. To solve the problem of complex gel preparation and complicated transportation, the novel gel is compressed, pelletized, and dried into expanded particles through industrial granulation, and the disadvantage of too fast expansion of expanded particles is optimized through physical film coating treatment. Finally, a novel nanoparticle coupling expanded granule plugging agent is developed. The granules have the characteristics of slow expansion.
3. Evaluation of the performance of the novel nanoparticle coupling expanded granule plugging agent. After 30 days of high temperature and high salt aging conditions, the granule expansion performance is good, and the granules have high strength and good long-term stability performance. With the increase of temperature or salinity, the expansion ratio of particles shows a decreasing trend, indicating that excessive temperature and salinity inhibit the water absorption ability of particles. The water plugging rate of particle plugging agents is as high as 97.84%, which is superior to other widely used particle-based plugging agents.

## 4. Materials and Methods

### 4.1. Experimental Materials

Experimental water: Simulated formation water prepared according to the ion composition of the produced water from the Tahe Oilfield, as shown in Table 3.

Experimental chemicals: hydroquinone, thiourea, hexamethylene tetramine, nano zirconium dioxide, nano silicon dioxide, nano aluminum oxide, nano iron oxide, nano zinc oxide, nano magnesium oxide, nano titanium dioxide, nano calcium carbonate, attapulgite soils, shown in Table 4. All drugs were analytically pure and provided by Shanghai Macklin Biochemical Technology Co., Ltd., Shanghai, China. The polymers used in this paper is Acrylamide/2-acrylamido-2-methylpropanesulfonic copolymer salt, belongs to block copolymers, with a solid content of >88%, AMPS content of 58%, and viscosity average molecular weight of 5 million; polymers were purchased from Shandong Baomo Biochemical Co., Ltd. (Dongying, China).

### 4.2. Experimental Apparatus

FT-IR Hoffen-10, Shanghai ZHUJIN Analytical Instrument Co., Ltd., Shanghai, China; EM-30AX scanning electron microscope (SEM), COXEM, Korea; DV2T Viscometer, Brookfield, WI, USA (belongs to the plate/cone type); precision aging tank (6-bore screws, 500 mL volume), Jiangsu Lianyou Scientific Research Instrument Co., Ltd., Nantong, China; 85–2 thermostatic magnetic stirrer, Changzhou Longhe Instrument Manufacturing Co., Ltd., Changzhou, China; Core Flow System Device, Jiangsu Lianyou Scientific Research Instrument Co., Ltd., Nantong, China.

### 4.3. Experimental Methods

#### 4.3.1. Determination of Gel Dehydration Rate

Put the prepared gelling solution into an ampoule bottle, which is recorded as m_1_; After gelling, take it out of the incubator, open the ampoule, and weigh the mass of gel dehydrated water, which is recorded as m_1_. The ratio of this mass to the mass of the initial gelling solution is the dehydration rate. Dehydration rate = (m_1_/m) × 100%.

#### 4.3.2. Determination of Expansion Multiplier

Characterize the water absorption and swelling performance of the granules by the mass multiplier of water absorption of the granules: weigh the mass of the dried granules m_0_, add them into the simulated stratigraphic water of Tahe, stir them into a dispersion system, then filter the granule solution through a filter, absorb the free water on the surface of the granules with filter paper, weigh the mass of the granules m_1_ after water absorption and swelling, and the swelling multiplier is S_w_ = m_1_/m_0_.

#### 4.3.3. Determination of Granule Strength

The *method* of foreign scholar J. E. Smith [17] was used to determine the granule strength. The experimental steps are as follows: (1) Screen out the granules with a granule size of 0.4–0.6 mm, and age the granules under the set high temperature and high salt conditions; (2) Prepare the aged granules into a granule suspension with a mass concentration of 10%; (3) Install a 10-mesh screen at the outlet of the sand-filling tube and drive the granule suspension at a high flow rate of 25 mL/min until it completely flows out of the sand-filling tube, and record the first differential pressure P_1_; (4) Collect the outgoing suspension and flow through the screen again under the same conditions, and record the second differential pressure P_2_. Toughness Index = P_1_/P_2_.

#### 4.3.4. Measurement of Particle Blocking Rate in Core Flow Experiment

(1) Saturate the core with formation water, raise the temperature to 130 °C, establish bound water saturation through oil flooding, and let it stand for 24 h; (2) Simulate the displacement of formation water to 98% water content in the core, record the pressure after liquid production, and calculate the K_W_ before plugging agent injection; (3) Reverse injection of a 10% particle solution 1 PV and aging at 130 °C for a while; (4) Forward water drive again, record the breakthrough pressure and calculate the K_w_’ after core plugging after the pressure stabilizes.

## Figures and Tables

**Table 1:** Effect of different types of nanoparticles on the dehydration rate of gel.

20 Days	Nanoparticle ZrO_2_	Nanoparticle SiO_2_	Nanoparticle Al_2_O_3_	Nanoparticle Fe_2_O_3_	Nanoparticle ZnO	Nanoparticle MgO	Nanoparticle TiO_2_	Nanoparticle CaCO_3_	Attapulgite Soil
Gel viscosity, Pa·s	36.8	43	37	42.8	36	36.4	19.8	20.6	27.2
Dehydration rate%, %	8.4	2.8	10.2	4.3	9	12.5	36.2	30.8	24

**Table 2:** Toughness Index of Granules at Different Aging Times.

Times	2 h	1 Day	2 Days	5 Days	10 Days	20 Days	30 Days
P_1_/MPa	0.06	0.058	0.069	0.072	0.07	0.074	0.074
P_2_/MPa	0.045	0.051	0.056	0.051	0.046	0.047	0.045
Toughness index	1.33	1.14	1.23	1.41	1.52	1.57	1.61

**Table 3:** Simulated formation water ion composition.

Ion Content/(mg·L^−1^)					Total Salinity /(mg·L^−1^)	pH Value
Cl^−^	HCO_3_^−^	Ca^2+^	Mg^2+^	Na^+^ + K^+^		
138,000	200	11,000	1500	73,000	223,700	6.8

**Table 4:** Nanoparticle properties.

	Nanoparticle ZrO_2_	Nanoparticle SiO_2_	Nanoparticle Al_2_O_3_	Nanoparticle Fe_2_O_3_	Nanoparticle ZnO	Nanoparticle MgO	Nanoparticle TiO_2_	Nanoparticle CaCO_3_	Attapulgite Soil
Particle size, nm	25 ± 5	20 ± 5	30 ± 5	30	30 ± 5	30	25	30	30 ± 5
Surface Area (m^2^/g Gsurface)	150–500	45–150	500–700	400	500–700	450	150	450	500–800

## Data Availability

No new data were created.
